# Investigation of the relationship between sleep-related parameters and metabolic syndrome (MetS) among youths in the Southeast of Iran

**DOI:** 10.1186/s13098-023-01072-3

**Published:** 2023-05-05

**Authors:** Majid Kazemi, Parvin Khalili, Mahsa Kazemi, Hadi Hasani, Marjan Sadeghi, Zahra Jamali

**Affiliations:** 1 Department of Medical Surgical Nursing, Faculty of Nursing and Midwifery, Non- Communicable Disease Research Center, Rafsanjan University of Medical Sciences, Rafsanjan, Iran; 2 Department of Epidemiology, School of Public Health, Social Determinants of Health Research Center, Rafsanjan University of Medical Sciences, Rafsanjan, Iran; 3 Department of Neurology, School of Medicine, Kerman University of Medical Sciences, Kerman, Iran; 4 Department of Medical Surgical Nursing, Jovein School of Nursing, Sabzevar University of Medical Sciences, Sabzevar, Iran; 5 Non-Communicable Diseases Research Center, Rafsanjan University of Medical Sciences, Rafsanjan, Iran; 6 Clinical Research Development Unit (CRDU), Niknafs Hospital, Rafsanjan University of Medical Sciences, Rafsanjan, Iran

**Keywords:** Metabolic syndrome, Sleep-related parameters, Youths, Prospective Epidemiological Research Studies in Iran (PERSIAN), Rafsanjan Cohort Study, Sleep duration

## Abstract

**Background and aim:**

There are few studies and inconsistent findings on the role of sleep-related parameters in the development of metabolic syndrome (MetS) among youths. In this study, we aim to investigate the relationship between sleep-related parameters and MetS among youths in a large sample size in Rafsanjan, a region in the southeast of Iran.

**Methods:**

The current cross-sectional study was performed on 3,006 young adults aged 15–35, who registered for Rafsanjan Youth Cohort Study (RYCS), as part of Rafsanjan Cohort Study (RCS)). In fact, RCS is a branch of the prospective epidemiological research studies in Iran (PERSIAN). In the present study, we included 2,867 youths after excluding some subjects with missing information on MetS components. MetS was diagnosed based on Adult Treatment Panel III (ATP III) criteria. Besides, data on sleep-related parameters were collected by self-report questionnaires.

**Results:**

The overall prevalence of MetS was 7.74% among the participants. In addition, bedtime, wake time, napping, night shift work, and sleep duration per night and day had no association with the higher odds of having MetS. In contrast, long sleep duration at night was associated with the lower odds of high waist circumference (WC) (OR: 0.82,95% CI :0.67–0.99).

**Conclusion:**

In the present study, long sleep duration at night was associated with lower odds of central obesity. However, more longitudinal studies with the objective measurement of sleep-related parameters are needed to verify the associations reported in the current study.

**Supplementary Information:**

The online version contains supplementary material available at 10.1186/s13098-023-01072-3.

## Introduction

Metabolic syndrome (MetS), also named syndrome X or insulin resistance syndrome, is a condition in which a group of risk factors come together and provide high susceptibility to some diseases, such as diabetes, coronary heart disease, fatty liver, and some cancers [1]. According to Adult Treatment Panel III (ATP III) criteria, having at least three conditions among high triglyceride (TG), elevated fasting blood sugar (FBS), decreased high density lipoprotein cholesterol (HDL), high blood pressure (BP), and high waist circumference (WC) is enough for being diagnosed with MetS [2].

This disease is highly prevalent among populations across the world and affects both genders. In different studies, the prevalence of MetS has been reported at 20 to 25% in male adults [3, 4], and about 0-19.2% in children [5]. The prevalence of this syndrome is about 80% in type 2 diabetes; besides, it is about 25.9 and 22.5% in females and males, respectively, with type 1 diabetes [6].

This disorder can cause a two-fold and a four-fold increased risk of CVDs and stroke, respectively. Besides, it increases the future probability of type 2 diabetes by 5 times, accompanied by a higher risk of certain cancers. It is suggested that high-calorie food intake, sedentary lifestyle, and being overweight increase the incidence of MetS [1, 7, 8]. In contrast, an improvement can be achieved by changing the lifestyle, increasing daily activity, modifying the consumption of high-calorie foods, reducing weight, and taking lipid-lowering drugs under medical supervision [1].

Past research shows that metabolic disturbances in our body can be associated with sleep deficiency, insomnia, and narcolepsy [9, 10]. In fact, sleep is coordinated by the circadian clock that is a regulatory system within our body that synchronizes different activities, including sleep and metabolic functions; thus, it goes without saying that sleep and MetS are related factors [11]. However, the biological clock can lose its normal balanced function by some detrimental behavioral routines, like improper sleep habits, low physical activity, and increased high-calorie food intake [12]. Sleep disturbances are stated to affect several factors in the body, including energy homeostasis, risks of inflammation, impaired glucose tolerance, insulin resistance, and obesity, all of which contributing to MetS; nonetheless, it is still unclear which sleep parameters and habits are associated with MetS [12].

There were some inconsistent results in the literature review. Accordingly, some studies found that short sleep durations were associated with MetS [13–16], yet some others concluded that short sleep durations were not related to it [17]. Najafian et al. reported that while short sleep durations could be associated with MetS, longer sleep durations had a protective effect on developing MetS in the future [16]. Qian et al. reported that both short and long sleep durations could increase the risk of MetS [18]. In fact, these inconsistencies are observed in meta-analysis articles as well [14, 19].

There are few studies and inconsistent findings on the role of sleep in the development of MetS among youths. To the best of our knowledge, there is one study in Iran to have reported that the sleep duration of less than 8 h per day can result in a higher risk of MetS in schoolchildren aged 7 to 18 [20]. Against this backdrop, we conducted the present study to determine the relationship between sleep-related parameters and MetS among youths of a large sample size in Rafsanjan City in the southeast of Iran.

## Materials and methods

### Study population

The current cross-sectional study was performed on 3,006 young adults aged 15–35, who registered for Rafsanjan Youth Cohort Study (RYCS), as part of Rafsanjan Cohort Study (RCS)). RCS is a branch of the prospective epidemiological research studies in Iran (PERSIAN) [22]. RYCS was started in 2016 in both urban and rural areas of Rafsanjan City. In fact, follow-up visits are still in progress. In the present study, we included 2,867 out of 3,006 participants from the baseline phase of RYCS after exclusion of those subjects with missing information on MetS components.

All procedures of invitation, interview, measurement, and physical examinations were followed in compliance with the PERSIAN cohort protocols [22]. All the questionnaires used in this study were prepared as a software program, and the interviews were conducted face-to-face; additionally, individual responses were entered directly into the software by the interviewers. The questionnaires were validated in the PERSIAN cohort study [22]. In the baseline phase of data collection, a comprehensive questionnaire with demographic information on personal habits, such as smoking, substance use, and the like, medical history, and sleep habits was designed by trained interviewers, with blood samples collected.

### Laboratory assessment

All the subjects were asked to fast for at least 12 h before the examination. Blood samples were taken between 7:00 AM and 9:00 AM. In addition, fasting blood sugar (FBS), total cholesterol, HDL cholesterol, LDL cholesterol, and TG were measured by a Biotechnia analyzer (BT 1500, Italy) at the Central Laboratory of the cohort center.

### MetS assessment

Metabolic syndrome was defined when three or more of the following findings were achieved according to NCEP ATP III criteria:

1. Waist circumference ≥ 102 cm in men and ≥ 88 cm in women.
2. TG ≥ 150 mg/dl or use of medications for lowering TG.
3. HDL < 40 mg/dl in men or < 50 mg/dl in women or use of medications.
4. Hypertension (systolic blood pressure ≥ 130 mmHg or diastolic pressure ≥ 85 mmHg) or use of antihypertensive medications.
5. Hyperglycemia (FBS ≥ 100 mg/dl or use of anti-hyperglycemic medications) [23].

In the present study, subjects with 3 positive criteria out of 5 metabolic syndrome criteria were considered having MetS even if the other 2 criteria were missing. In addition, subjects with 3 negative criteria were considered the non-MetS group even if the other 2 criteria were missing.

### Sleep parameters assessment

The population’s sleep habits were assessed using a self-report questionnaire. Information on sleep habits included bedtime, wake time, daytime napping, and night shift work.

In the present study, sleep duration at night included the time from the bedtime to the wake time in the morning. The subjects were classified into three groups, according to their sleep duration at night. In this regard, for the subjects aged 15–18, sleep duration was considered short (< 8 h), normal (8–10 h), and long ≥ 11 h. On the other side, for the subjects aged over 18, sleep duration was considered short (< 6 h), normal (6–8 h), and long ≥ 9 h) [13, 24].

In fact, sleep duration per day was the sum of sleep duration at night and daytime napping hours. Sleep duration per day was classified into the 4 groups of < 6 h, 6–7 h, 8–9 h, and ≥ 10 h [9, 15]. In the multivariable analysis, the 8–9 h category was selected as the reference.

### Assessment of other variables

Blood pressure and anthropometric measurements were performed by trained health professionals.

In terms of age, the subjects were divided into three groups according to the 25th and 75th percentiles (≤ 20, 21–30, and ≥ 30 years).

In addition, education levels were divided into ≤ 12 years and > 12 years based on the median. Besides, the BMI was classified into 3 groups (< 25, 25-29.9, and ≥ 30 kg/m²).

Physical activity scores were evaluated based on weekly physical activity using a two-item questionnaire. The participants were asked about alcohol consumption, hookah smoking, and cigarette smoking. The questionnaires (sleep parameters, physical activity, and personal habits) used in this study were part of the PERSIAN cohort study questionnaires, which have been provided in the supplementary materials (supplementary document 1).

It is worth noting that the frequency difference between the total number and some of the covariates was related to the missing data.

### Data analysis

To describe the data, frequency (%) and mean were used for categorical variables (SD: standard deviation) and quantitative variables, respectively.

The individuals’ baseline characteristics were compared between the groups of our study (non- MetS and MetS) using a chi-square test (χ²) and a t-test for categorical and continuous variables, respectively. In addition, we used binary logistic analysis to determine the odds ratios (ORs) and the corresponding 95% confidence intervals (CI) for the relationship between MetS and sleep-related parameters. Besides, we used crude and adjusted models in the regression analysis. Potential confounding parameters were recognized based on subject matter knowledge and relevant epidemiological literature. Next, they were entered into the models sequentially according to their hypothesized strengths of association with sleep-related parameters and MetS. Variables with a p-value < 0.25 were considered confounders. The baseline (crude) model was stratified based on sleep-related parameters. In addition, the adjusted model was adjusted for confounding variables, including age (continuous variable), gender (male/female), education years (continuous variable), cigarette smoking (yes/no), alcohol consumption (yes/no), hookah smoking (yes/no), and physical activity score (continuous variable). All analyses were performed in Stata 14. All p-values were two-sided, with p-values < 0.05 and 95% confidence intervals considered statistically significant.

## Results

**Fig. 1:**
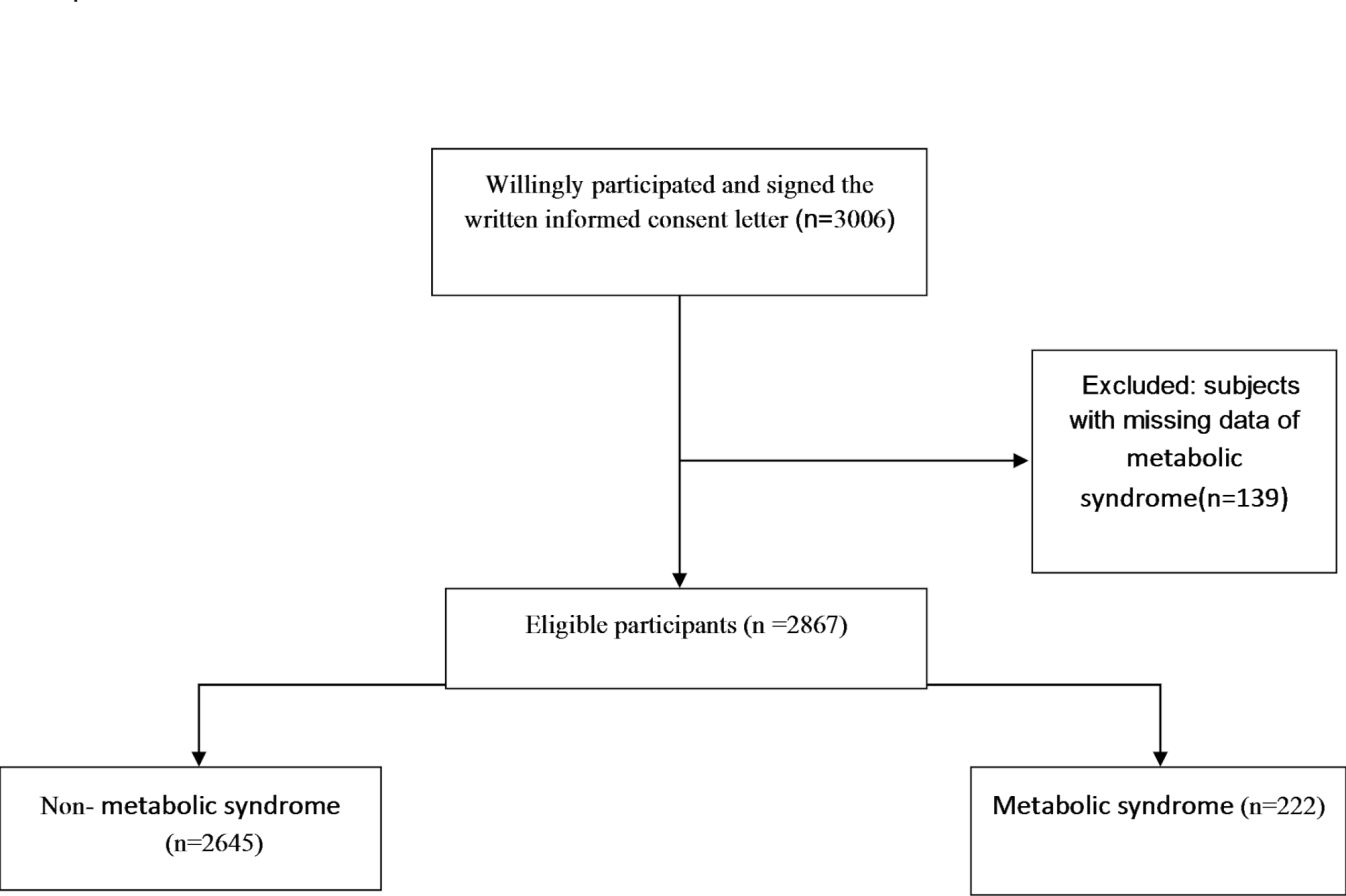
Flow chart of the study design of metabolic syndrome in Rafsanjan Youth Cohort Study

Figure 1 shows the flow chart of the study design of MetS among youths in RYCS. A total of 2,867 subjects from the base-line phase of RYCS, who had completed information on MetS, were included. The overall prevalence of MetS was 7.74%, according to NCEP-ATP III criteria. High WC had the highest frequency rate (31.32%) among MetS components. Next, elevated TG (26.54%), elevated FBS (23.26%), low HDL (12.85%), and elevated BP (3.99%) were the MetS components of the highest frequency.

Table 1 shows the study participants’ sociodemographic features, personal habits, and sleep parameters. In fact, the prevalence of MetS was significantly higher in women than in men (9.75% and 5.14%, respectively). In addition, subjects with MetS had a significantly higher mean age than those without MetS (P < 0.05).

**Table 1:** Demographic characteristics of study participants (n = 2867)

Characteristics	All (n = 2867)	Non-MetS (n = 2645)	MetS (n = 222)	P-Value
**Age - yr. no. (%)**				
≤ 20	722 (25.19%)	700 (96.95%)	22 (3.05%)	0.000
21–30	1258 (43.89%)	1161 (92.29%)	97 (7.71%)	
≥ 30	886 (30.91%)	783 (88.37%)	103 (11.63%)	
Mean ± SD	25.82 ± 6.07	25.59 ± 6.10	28.60 ± 4.97	0.000
**Gender - no. (%)**				
Female	1621(56.54%)	1463 (90.25%)	158 (9.75%)	0.000
Male	1246 (43.46%)	1182 (94.86%)	64 (5.14%)	
**Education.years- no. (%)**				
≤ 12 years	1770 (61.80%)	1622 (91.64%)	148 (8.36%)	0.120
≥ 13 years	1094 (38.20%)	1020(93.24%)	74 (6.76%)	
Mean ± SD	12.31 ± 3.01	12.33 ± 3.01	11.97 ± 3.10	0.086
**Physical activity score - no. (%)**				
Median(IQR)	0(0-120)	0(0-120)	0(0–90)	0.113
**BMI - no. (%)**				
< 25	1595 (55.77%)	1576 (98.81%)	19 (1.19%)	0.000
25-29.9	816 (28.53%)	731 (89.58%)	85 (10.42%)	
≥ 30	449 (15.70%)	331 (73.72%)	118 (26.28%)	
Mean ± SD	26.16 ± 53.62	25.74 ± 55.73	31.19 ± 10.22	0.145
**Alcohol consumption - no. (%)**				
Yes	688 (24.00%)	648 (94.19%)	40 (5.81%)	0.030
No	2179 (76.00%)	1997(91.65%)	182 (8.35%)	
**Cigarette smoking - no. (%)**				
Yes	828 (28.88%)	773 (93.36%)	55 (6.64%)	0.160
No	2039 (71.12%)	1872 (91.81%)	167 (8.19%)	
**Hookah consumption - no. (%)**				
Yes	1474 (51.43%)	1378 (93.49%)	96 (6.51%)	0.013
No	1392 (48.57%)	1266 (90.95%)	126 (9.05%)	
**Bedtime**				
Before or on 12:00 am	2028 (70.74%)	1869 (92.16%)	159 (7.84%)	0.763
After 12:00 am	839 (29.26%)	776 (92.49%)	63 (7.51%)	
**Wake time**				
Before or on 6:00 am	653 (22.78%)	604 (92.50%)	49 (7.50%)	0.794
After 6:00 am	2214 (77.22%)	2041 (92.19%)	173 (7.81%)	
**napping**				
Yes	1162 (40.53%)	1071 (92.17%)	91 (7.83%)	0.884
No	1705 (59.47%)	1574 (92.32%)	131 (7.68%)	
**Night shift work**				
Yes	150 (5.23%)	145 (96.67%)	5 (3.33%)	0.038
No	2717 (94.77%)	2500 (92.01%)	217 (7.99%)	
**Sleep duration per night**				
Short	317 (11.06%)	302 (95.27%)	15 (4.73%)	0.104
Normal	1888 (65.85%)	1735 (91.90%)	153 (8.10%)	
Long	662 (23.09%)	608 (91.84%)	54 (8.16%)	
Mean ± SD	7.65 ± 1.44	7.65 ± 1.45	7.63 ± 1.34	0.822
**Sleep duration per day**				
< 6	99 (3.45%)	91 (91.92%)	8 (8.08%)	0.321
6–7	682 (23.79%)	631 (92.52%)	51 (7.48%)	
8–9	1387 (48.38%)	1268 (91.42%)	119 (8.58%)	
≥ 10	699 (24.38%)	655 (93.71%)	44 (6.29%)	
Mean ± SD	8.53 ± 1.71	8.53 ± 1.72	8.42 ± 1.52	0.330

Abbreviation: BMI: body mass index

The two groups had statistically significant differences in alcohol consumption, hookah smoking, night shift work (P = 0.030, 0.013, 0.038, respectively). However, there were no significant differences between MetS and non-MetS groups in terms of the BMI, education level, physical activity score, cigarette smoking, bedtime, wake time, napping, sleep duration per night, and sleep duration per day (P > 0.05).

Table 2 shows associations between sleep parameters and MetS and its components among the study participants, using the crude and adjusted models.

**Table 2:** Association of sleep parameters with metabolic syndrome and its components among study participants

		MetS	Central obesity	Elevated TG	Low HDL	Elevated FBS	Elevated BP
**Bedtime**							
After 12:00 am vs. before or on 12:00 am	Crude	0.95 (0.70–1.29)	0.73 (0.61–0.87)	1.06 (0.88–1.27)	0.94 (0.74–1.20)	0.84 (0.69–1.02)	0.98 (0.66–1.47)
	Adjusted*	1.16 (0.85–1.59)	0.88 (0.73–1.07)	1.14 (0.94–1.38)	0.98 (0.76–1.27)	0.94 (0.77–1.5)	1.11 (0.74–1.68)
**Wake time**							
After 6 am vs. before or on 6 am	Crude	1.04 (0.75–1.45)	0.88 (0.74–1.06)	0.82 (0.67–0.99)	1.16 (0.86–1.51)	0.89 (0.72–1.09)	1.16 (0.73–1.83)
	Adjusted*	1.32 (0.93–1.86)	1.00 (0.81–1.23)	1.08 (0.87–1.33)	0.98 (0.73–1.30)	1.14 (0.92–1.42)	1.55 (0.96–2.49)
**Napping**							
Yes vs. no	Crude	1.02 (0.77–1.35)	0.85 (0.73–0.99)	0.94 (0.79–1.11)	0.91 (0.72–1.13)	1.05 (0.88–1.26)	1.27 (0.88–1.83)
	Adjusted*	1.05 (0.79–1.40)	0.91 (0.77–1.09)	0.90 (0.76–1.08)	0.98 (0.78–1.24)	1.05 (0.88–1.26)	1.26 (0.87–1.83)
**Night shift work**							
Yes vs. no	Crude	0.40 (0.16–0.98)	0.45 (0.30–0.69)	2.42 (1.74–3.38)	0.18 (0.07–0.48)	1.30 (0.90–1.88)	0.94 (0.41–2.16)
	Adjusted*	0.45 (0.18–1.15)	0.93 (0.59–1.47)	1.29 (0.90–1.83)	0.61 (0.21–1.71)	0.88 (0.60–1.30)	0.63 (0.26–1.50)
**Sleep duration per night**							
Short vs. normal	Crude	0.56 (0.33–0.97)	0.63 (0.48–0.82)	1.01 (0.78–1.32)	0.77 (0.52–1.15)	0.80 (0.60–1.08)	0.80 (0.42–1.52)
	Adjusted*	0.75 (0.43–1.31)	0.89 (0.66–1.20)	1.20 (0.90–1.59)	0.76 (0.50–1.17)	0.96 (0.71–1.31)	0.96 (0.50–1.84)
Long vs. Normal	Crude	1.00 (0.73–1.39)	1.04 (0.87–1.25)	0.79 (0.64–0.97)	1.52 (1.19–1.94)	0.94 (0.76–1.16)	0.99 (0.64–1.53)
	Adjusted*	0.94 (0.68–1.31)	0.82 (0.67–0.99)	0.97 (0.78–1.21)	1.18 (0.92–1.52)	1.04 (0.84–1.29)	1.09 (0.70–1.71)
**Sleep duration per day**							
< 6 h vs. 8–9 h	Crude	0.94 (0.44–1.98)	0.73 (0.47–1.15)	1.63 (1.07–2.49)	0.42 (0.18–0.97)	0.81 (0.49–1.32)	1.40 (0.59–3.31)
	Adjusted*	0.89 (0.41–1.91)	0.79 (0.48–1.31)	1.32 (0.85–2.06)	0.52 (0.22–1.24)	0.69(0.42–1.15)	1.20 (0.50–2.88)
6–7 h vs. 8–9 h	Crude	0.86 (0.91–1.21)	1.00 (0.82–1.21)	1.06 (0.87–1.31)	0.73 (0.54–0.98)	0.92 (0.74–1.14)	1.07 (0.68–1.66)
	Adjusted*	0.84 (0.60–1.20)	1.06 (0.86–1.31)	0.94 (0.76–1.17)	0.79(0.58–1.07)	0.84 (0.68–1.05)	0.98 (0.63–1.54)
≥ 10 h vs. 8-9 h	Crude	0.72 (0.50–1.02)	0.90 (0.74–1.09)	0.82 (0.66–1.01)	1.20 (0.93–1.55)	0.74 (0.59–0.92)	0.71 (0.43–1.17)
	Adjusted*	0.75 (0.52–1.09)	0.85 (0.69–1.06)	1.03 (0.83–1.29)	0.98 (0.75–1.29)	0.86 (0.68–1.08)	0.81 (0.49–1.35)

Abbreviations: MetS: Metabolic syndrome, TG: Triglyceride, HDL: high-density lipoprotein, FBS: Fasting blood sugar, BP: blood pressure.

*) adjusted for confounding variables age (continuous variable), gender (male/female), education years (continuous variable), cigarette smoking (yes/no), alcohol drinking (yes/no), hookah consumption (yes/no), and physical activity score (continuous variable).

Accordingly, high WC was associated with sleeping after 12:00 PM at night, napping, and night shift work in the crude analysis. However, these associations were not observed later in the adjusted model. In the adjusted model, long sleep duration at night decreased the odds of central obesity by about 19% (OR: 0.82, 95% CI :0.67–0.99) compared to the reference group.

In crude analysis, waking up after 6:00 AM in the morning and long sleep duration at night played a protective role in high TG. In addition, having night shift work, short sleep duration at night, and sleep duration of < 6 h per day played the role of a risk factor for high TG; however, these associations were not observed any longer after adjusting the confounding variables.

On the other side, regarding crude analysis, having night shift work, sleep duration of < 6 h per day, and sleep duration of 6–7 h per day decreased the odds of low HDL. Besides, long sleep duration at night increased the odds of low HDL compared to the reference group. However, these associations were not observed after adjusting the confounding variables. In addition, elevated FBS and BP did not show any associations with sleep-related parameters.

## Discussion

Evidence regarding associations between sleep parameters and MetS among youths is sparse. To the best of our knowledge, this study is the first one to have reported associations between MetS and sleep parameters among youths in a large population-based cohort study in Iran. This cross-sectional study showed a significant association between the odds of MetS, high WC, elevated TG, and low HDL with some sleep parameters, such as night shift work, sleeping after 12:00 PM, napping, long sleep duration at night, short sleep duration at night, waking up after 6:00 AM, sleep duration of 6–7 h per day, and sleep duration of ≥ 10 h per day. However, when these analyses were adjusted for more confounding variables, such as age, gender, education, cigarette smoking, alcohol consumption, hookah smoking, and physical activity, no such significant associations were observed. In fact, there was only a significant association between long sleep duration at night and the lower odds of high WC after adjustment for confounding variables. In addition, we found that these confounding variables should be considered as potential confounders for the analysis of associations between MetS and its components with sleep parameters.

Sleep is a crucial factor for health, yet its role in causing different diseases and its healthy duration are still debated. On June 29, 2022, the American Heart Association (AHA) added sleep duration to its cardiovascular health checklist. Next, the previous checklist called “Life’s Simple 7” was named “Life’s Essential 8” because of the importance of “healthy sleep” [25]. Some studies report that long sleep duration is associated with an increased risk of cancer incidence and cancer mortality [26]. On the other hand, other studies suggest that short sleep duration (≤ 5–6 h) and daytime napping are associated with dementia, all-cause mortality (27–29), and a higher BMI in adults [30]. In contrast, a new study conducted on mice found that the relationship between sleep duration and Alzheimer’s disease was caused by some rare genes that both human and mouse populations can have, which help them sleep less than usual while being alert [31]. This study suggests that sleep duration is not a direct risk factor for some diseases, yet sleep quality may play a stronger role than sleep duration.

There are a lot of inconsistencies about the relationship between short sleep duration and MetS in past research. Some studies report that short sleep duration is related to MetS [13–16], yet some others conclude that short sleep duration is not connected to MetS [17]. Moreover, some studies have reported that long sleep duration can decrease the risk of MetS [16], while others reject this hypothesis and report that long sleep duration is not related to MetS [13, 14, 32, 33]. On the other hand, some studies have reported that both short and long sleep durations can increase the risk of MetS [9, 18, 19, 32, 34]. These different results can be due to differences in the diet, daily life stress, study design (cross-sectional or longitudinal), sample sizes, analytic strategies, participants’ characteristics, ethnicity, diagnostic criteria for metabolic syndrome, location, type of study (community-based or hospital-based), sleep measurement, and differences in the adjusted confounding factors.

The present study found no relationship between sleep duration and the odds of MetS. The lack of any relationship between MetS and sleep parameters in the present study could be due to the low prevalence of MetS in the study population. Another reason could be that sleep quality may play a stronger role in developing MetS than sleep duration, napping, wake time, and bedtime.

Consistent with our findings, a recent cohort study conducted in Japan with 3,880 participants found no significant relationship between bedtime and sleep duration with MetS, suggesting that irregular sleep could be a stronger variable for MetS than bedtime and sleep duration [17]. Another study on Korean adolescents showed that the odds of MetS were not associated with sleep duration [35]. In a cross-sectional study on 1,079 residents of Qazvin City with the mean age of 40.08, wake time, sleep duration, and bedtime were not associated with MetS, yet sleep disturbances were associated with an increased risk of MetS [24]. In a study on 4,579 old Chinese adults, both long and short sleep durations, daytime napping, and wake up time of ≤ 6 were associated with a higher risk of MetS. However, no significant differences were observed between adults with MetS and those without non-MetS in terms of self-reported sleep quality [18].

Consistent with the findings of the present study, long sleep duration was not associated with MetS and its components, based on multiple logistic regression in the study of Wu et al. on 7,300 adults in Taiwan. In that study, short sleep duration was positively associated with MetS and hyperglycemia but not with other MetS components [13]. In the study of Kim et al. on 133,608 Koreans within the age range of 40–69, after adjusting for covariates, sleep duration per day was associated with MetS, elevated WC, elevated TG, low HDL, and elevated FBS, but not with elevated BP [9]. The inconsistencies with the present study could be due to differences in the sample size (133,608 versus 2,867), age range (40–69 versus 15–35), ethnicity (Koreans versus Iranian), and adjusted confounding factors. In fact, the Korean study was adjusted for further confounding variables, such as menopausal status and dietary intake.

Among the components of MetS, we found that individuals with long sleep duration at night were by 0.81 times less likely to have central obesity. In the study by Tasali et al., additional sleep duration was associated with the increased feeling of vigor and decreased desire for sweet and salty foods by 62%, without affecting the desire for fruits, vegetables, and protein-rich foods [36]. In addition, in a longitudinal analysis of 293 participants aged 18–65, the extension of sleep duration was associated with decreased visceral adipose tissue in multivariable analysis [37]. In another study by Haines et al. on preschoolers, the authors reported that increased sleep duration could be effective in reducing the BMI [38]. However, there is no clear reason why longer sleep duration is associated with lower central obesity. This could be explained by some neuroendocrine alterations [39]. In some studies, in response to experimental sleep restrictions, leptin resistance, decreased leptin (satiety hormone), increased ghrelin (appetite-stimulating hormone) [12], reduced peptide YY (an inhibitor of food intake) [40], and elevated cortisol in the afternoon or evening [41] were reported. According to the authors, these neuroendocrine alterations appeared to promote weight gain in the sleep restriction mode despite unchanged or even increased energy consumption [12]. On the other hand, past research showed that short sleep duration was associated with higher caloric intake and lower voluntary physical activity [42]. In contrast, long sleep possibly helps control appetite; in addition, it may help individuals make healthier food choices and increase daily activity, thereby reducing overall obesity as well as WC.

Consistent with the current results, a follow-up study on Brazilian adolescents reported that the time-in-bed reduction was not associated with changes in WC [43]. Another cross-sectional study on obese American children and adolescents showed that sleep duration was not associated with the risk of MetS, adiposity, and insulin resistance [44]. However, some prospective cohort studies have shown that short sleep duration is associated with the risk of high WC in children [45, 46]. A cross-sectional study was conducted on 3,843 children and adolescents aged 7–18 from 30 Iranian provinces. Accordingly, the association of short sleep duration and bedtime with MetS components was not statistically significant in the multivariate model, being consistent with our findings; however, short sleepers had higher odds of MetS and high BP in that study [20]. The differences in findings could be explained by differences in the age range, diagnostic criteria for metabolic syndrome, and definitions of sleep duration. Unlike the present study, short sleep duration in the aforementioned study was considered as sleep duration ≤ 8 h per day. The main strength of the present study was its large sample size with extensive information on potential confounders, such as demographic features, education, cigarette smoking, alcohol consumption, hookah smoking, and physical activity scores. On the other hand, data on blood chemistry and anthropometry were not based on self-reports. Besides, anthropometric measurements were performed by trained experts according to the PERSIAN cohort protocols, and biochemical metabolic parameters were measured by the RCS laboratory. However, there were some limitations in our study. Firstly, since sleep duration and quality can be influenced by the diet [47], dietary intake was not assessed in our study population. Secondly, due to cross-sectional design, we could not prove any cause-effect association between sleep-related parameters and MetS. It is recommended that future prospective cohort studies determine sleep-related parameters as a cause of MetS. In fact, it is possible that MetS-related changes cause sleep disturbances. Thirdly, information on sleep-related parameters was assessed using self-report questionnaires, instead of objective measurements, which might have caused an information bias. However, a study found that self-reported sleep parameters were reasonably valid as against objective measurements [48]. Fourthly, no comprehensive data on sleep quality were available for analysis. Fifthly, the covariates, including smoking cigarette, hookah smoking, and alcohol consumption were included in the final model as categorical variables. Given that cigarette smoking, hookah smoking, and alcohol consumption have a dose-response relationship with health outcomes, there may be residual confounding effects [9]. Finally, all the participants of our study were from an Iranian population, so the results cannot be generalized to other races and ethnic populations.

## Conclusion

In conclusion, bedtime, wake time, napping, having night shift work, sleep duration per night, and sleep duration per day showed no relationship with the higher odds of having MetS. In contrast, long sleep duration at night was associated with the lower odds of high WC in the present study. However, more longitudinal studies with the objective measurement of sleep-related parameters are needed to verify the associations reported in the current study.

## Electronic supplementary material

Below is the link to the electronic supplementary material.

Supplementary Material 1

## Data Availability

The data is not available publicly. However, upon a reasonable request, the data can be obtained from the correspondence.
