# Supplementary material for: Investigation of the relationship between sleep-related parameters and metabolic syndrome (MetS) among youths in the Southeast of Iran
Source: Diabetol Metab Syndr. 2023 May 5;15:91. doi: 10.1186/s13098-023-01072-3 (PMC10161496; doi:10.1186/s13098-023-01072-3)
Supplement: Supplementary file 1 — Supplementary Material 1 [file 13098_2023_1072_MOESM1_ESM.docx]

The questionnaires (sleep parameters, physical activity, and personal habits) used in this study were part of the PERSIAN cohort study questionnaires including:

**Sleep habits:**

Bedtime: what time do you usually fall asleep at night?

Wake time: what time do you usually wake up in the morning?

Napping: Apart from sleeping at night, do you also sleep during the day (three or more times a week)?

Night shift work: During the last month, have you worked a night shift (at least 6 hours between 9 pm and 6 am)?

**The physical activity:**

" During the past week, how many days did you exercise or do moderate to vigorous physical activity for at least 10 minutes?"

" During the past week, on days you did moderate to vigorous physical activity for at least 10 minutes, on average how many minutes a day did it take?".

**Personal habits**

Alcohol drinking: Have you ever consumed alcohol in your life?

Hookah consumption: Have you ever used hookah or pipe in your life?

Cigarette smoking: Have you ever smoked at least one whole cigarette in your life?

The answer was yes or no.
